# Tuning the degree of CO_2_ activation by carbon doping Cu_*n*_^−^ (*n* = 3–10) clusters: an IR spectroscopic study†

**DOI:** 10.1039/d2fd00128d

**Published:** 2022-11-03

**Authors:** Olga V. Lushchikova, Máté Szalay, Tibor Höltzl, Joost M. Bakker

**Affiliations:** a Radboud University, Institute for Molecules and Materials, FELIX Laboratory Toernooiveld 7 6525 ED Nijmegen The Netherlands joost.bakker@ru.nl; b Institut für Ionenphysik und Angewandte Physik, Universität Innsbruck Technikerstraße 25 6020 Innsbruck Austria; c MTA-BME Computation Driven Chemistry Research Group, Department of Inorganic and Analytical Chemistry, Budapest University ofTechnology and Economics Muegyetem rkp. 3 Budapest 1111 Hungary; d Furukawa Electric Institute of Technology Késmárk Utca 28/A 1158 Budapest Hungary

## Abstract

Copper clusters on carbide surfaces have shown a high catalytic activity towards methanol formation. To understand the interaction between CO_2_ and the catalytically active sites during this process and the role that carbon atoms could play in this, they are modeled by copper clusters, with carbon atoms incorporated. The formed clusters Cu_*n*_C_*m*_^−^ (*n* = 3–10, *m* = 1–2) are reacted with CO_2_ and investigated by IR multiple-photon dissociation (IR-MPD) spectroscopy to probe the degree of CO_2_ activation. IR spectra for the reaction products [Cu_*n*_C·CO_2_]^−^, (*n* = 6–10), and [Cu_*n*_C_2_·CO_2_]^−^, (*n* = 3–8) are compared to reference spectra recorded for products formed when reacting the same cluster sizes with CO, and with density functional theory (DFT) calculated spectra. The results reveal a size- and carbon load-dependent activation and dissociation of CO_2_. The complexes [Cu_*n*_C·CO_2_]^−^ with *n* = 6 and 10 show predominantly molecular activation of CO_2_, while those with *n* = 7–9 show only dissociative adsorption. The addition of the second carbon to the cluster leads to the exclusive molecular activation of the CO_2_ on all measured cluster sizes, except for Cu_5_C_2_^−^ where CO_2_ dissociates. Combining these findings with DFT calculations leads us to speculate that at lower carbon-to-metal ratios (CMRs), the C can act as an oxygen anchor facilitating the OC=O bond rupture, whereas at higher CMRs the carbon atoms increasingly attract negative charge, reducing the Cu cluster’s ability to donate electron density to CO_2_, and consequently its ability to activate CO_2_.

## Abstract 2

Carbon-doped anionic copper clusters exhibit a size- and dopant-loading dependent activity towards CO_2_, resulting in activation and dissociation.

## Graphical abstract

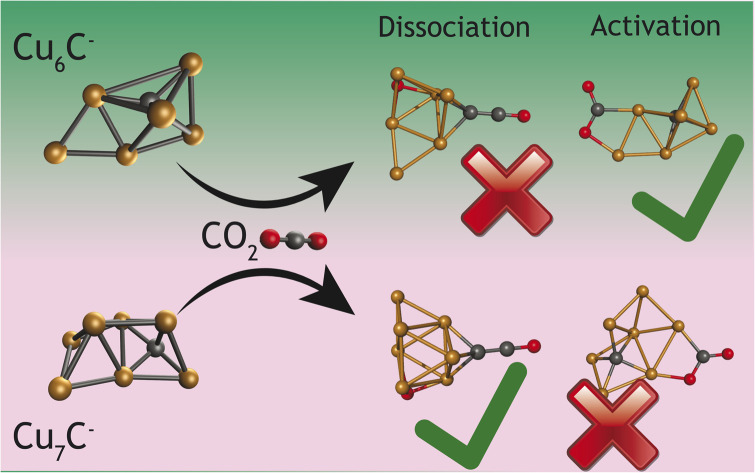

## Introduction

The continuous growth of carbon emissions has led, and continues to lead, to an accumulation of CO_2_ in the atmosphere, and the concomitant global warming. To mitigate this, an urgent solution is needed to reduce atmospheric CO_2_ concentrations. At the same time, CO_2_ is the largest carbon source on Earth, and it could be used as a feedstock for many valuable chemicals, such as alcohols and other higher hydrocarbons. The bottleneck lies in the high kinetic and thermal stability of the CO_2_ molecule, expressed in the C=O bond energy of more than 7 eV.^1^

One promising way for CO_2_ utilization is its hydrogenation to simple alcohols like methanol. Industrially this process takes place over a Cu/ZnO/Al_2_O_3_ catalyst at temperatures of 200–300 °C and pressures of 50–100 bar.^2^ The energy required to drive this reaction leads to additional CO_2_ emissions and elevated costs, while the one-pass selectivity towards methanol formation is rather low, with larger concentrations of CO and H_2_O formed. Thus, for direct CO_2_ conversion, a more selective catalyst is required. Many different catalyst materials have been tested to reduce reaction temperatures and increase the methanol yield.

One issue that seems not to affect relatively well-functioning CO_2_ hydrogenation catalysts is poisoning due to coke formation, a process that hampers the reverse reaction during methanol decomposition.^3^ Rather, metal carbide catalyst materials have shown enhanced activity in CO_2_ activation. This enhancement has been rationalized by increased catalyst stability upon adding carbon to the metal, and by the modification of the metal’s electronic and structural properties, changing its catalytic activity.^4,5^ Transition metal carbides (TMCs) have even demonstrated chemical activity similar, and sometimes even better, than that of the platinum group metals in the transformation of hydrocarbons and oxygen-containing molecules.^6–8^ TMCs are also considered to be promising catalysts for CO_2_ conversion by H_2_.^8^ However, experimentally, it was shown that, for example, Mo_2_C is quite aggressive and leads to CO_2_ dissociation, forming CO and H_2_O, *via* the reverse water gas shift (rWGS) reaction.^9,10^ Based on density functional theory (DFT) calculations, it is suggested that Mo_8_C_12_ nanoparticles are better catalysts, considering their high stability and moderate chemical activity in comparison to that of bulk Mo (too low) and Mo_2_C (too high).^11^ Similar conclusions were drawn for Ti carbides.^11^ Some transition metal catalysts with a carbon-to-metal ratio (CMR) below 1 easily break the C–O bond leading to CO formation.^6,7^ An increase in carbon content may enhance catalyst stability, it also leads to a decrease of the chemical activity, for instance observed for TiC and MoC, due to the ligand effect expressed in a downshift of the metal d-band center affecting the charge transfer from the metal to CO_2_ and, thus, also adsorption energy.^7,11–13^ Accordingly, the activity of a metal carbide catalyst towards CO_2_ dissociation can be tuned by varying the CMR of the catalyst.^14^

TMC catalytic activity can also be altered by the deposition of other metals on the TMC surface.^5,7,10,14^ Cu-promoted carbides exhibit enhanced selectivity towards methanol formation in comparison to both bare copper and bare TMC surfaces, like TaC, SiC, TiC, Mo_2_C, and MoC.^5,7,14^ It is also pointed out that the Cu–TMCs interface plays a crucial role. The hydrogenation of the reaction intermediates, such as HCO and H_2_CO, is energetically more favorable on the Cu sites, making small Cu clusters more suitable catalysts for methanol formation.^5,14^ Cu_4_/TiC has an even higher CO_2_ adsorption energy, and therefore methanol formation rate, than the commercial Cu/ZnO catalyst.^5,15,16^ However, most Cu/TMC catalyzed reactions proceed *via* CO formation and its subsequent hydrogenation, rather than *via* direct CO_2_ hydrogenation and a formate intermediate, as is the widely accepted reaction pathway over Cu catalysts.^2^ The high selectivity towards CO results in an elevated CO yield at the expense of methanol production. Therefore, it is important to understand what the direct influence of carbon is on the deposited clusters and how it influences CO_2_ activation.

The fine details of a reaction proceeding on the active sites can be studied at the molecular level using isolated clusters. Mass-selective gas-phase spectroscopy allows studying the interaction of CO_2_ with clusters of well-defined elemental composition. For example, photoelectron and IR spectroscopy have been used to study the interaction between CO_2_ and metal anions.^17–27^ This interaction can be characterized by four binding motifs: bidentate (η^2^), metal formate, oxalate, and dissociative adsorption. The variety in CO_2_ adsorption motifs is related to the difference in the degree of electron transfer from the metal center into the antibonding 2π_u_ LUMO of CO_2_.^28^ For Cu^−^ and other coinage metal ions in particular, it was found that CO_2_ binds in a formate-like fashion, where the metal ion replaces the hydrogen.^17,19^ The reaction of CO_2_ with isolated anionic metal clusters was also studied with flow tube reactor mass spectrometry,^29,30^ and IR spectroscopy.^31,32^ The nature of CO_2_ adsorption on these clusters appears to be size-dependent, for example on Pt_*n*_^−^ clusters, where Pt_4_^−^ shows CO_2_ activation, whereas Pt_5_^−^, Pt_6_^−^ and Pt_7_^−^ show dissociation.^31^ Another example is Co_*n*_^−^, where the Co^−^ ion binds two CO_2_ molecules in a bidentate configuration,^27^ and clusters with *n* > 7 dissociate CO_2_.^32^

In this work, we investigate how the carbon doping of anionic Cu clusters will influence the degree of CO_2_ activation, where the anionic charge state was chosen to facilitate electron transfer to the CO_2_ molecule. Anionic copper clusters containing 5–10 Cu atoms were doped with one or two C atoms and reacted with CO_2_. The formed [Cu_*n*_C_*m*_·CO_2_]^−^ species, with *n* = 3–10 and *m* = 1–2, were studied by mass-selective IR spectroscopy. The obtained spectra are compared to reference IR spectra of the clusters reacted with CO and with DFT calculated spectra.

## Methods

**Fig. 1:**
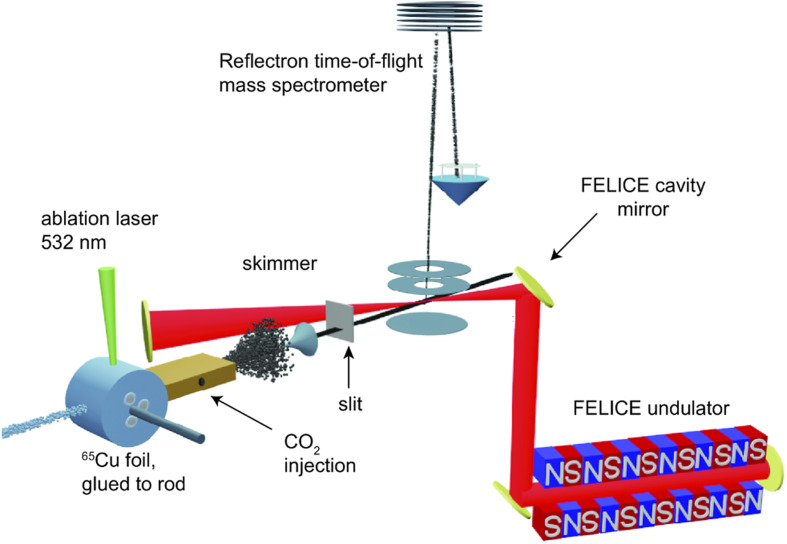
Schematic of experimental setup.

The experiment is shown schematically in Fig. 1. Carbon-doped anionic copper clusters Cu_*n*_C_*m*_^−^ are produced in a Smalley-type laser ablation source.^33^ An isotopically enriched Cu-65 (99.9%) target is ablated by the focused second harmonic of a Nd:YAG laser (532 nm, 14 mJ) in the presence of a He carrier gas that is injected by a pulsed valve (General Valve series 9, stagnation pressure 6 bar). Carbon-doped copper clusters were formed due to the presence of trace carbon in the carrier gas or residual pump oil vapour. The formed clusters are reacted with CO_2_ or CO by injecting the pure gases through a second pulsed valve with a 1 bar stagnation pressure into an extension of the clustering channel. The Cu_*n*_C_*m*_^−^ clusters react with CO_2_ or CO at room temperature in a reaction channel, forming [Cu_*n*_C_*m*_·CO_2_]^−^ or [Cu_*n*_C_*m*_·CO]^−^ complexes with *n* = 3–10, and *m* = 1–2. The bracket notation implies that no prior knowledge of the adsorption form of CO or CO_2_ is assumed. The gas mixture of helium and clusters is expanded into vacuum, forming a molecular beam that is collimated by a 2 mm diameter skimmer and an 8 × 2 mm slit aperture. The ions are then irradiated by IR laser light, and subsequently pulse-extracted by high voltage plates into an orthogonal time-of-flight mass spectrometer (TOF-MS), where they are mass separated and detected by a multichannel plate (MCP) detector. For the spectroscopic experiments, the molecular beam overlaps between the extraction plates at a 35° angle with the IR laser beam of the free-electron laser for intracavity experiments (FELICE).^34^ IR light is produced in the form of macropulses, consisting of an approximately 10 μs pulse train of transform-limited ps duration micropulses, and is characterized by a spectral bandwidth of ∼0.7% of the central frequency. The frequency range covered in this work is 320–2120 cm^−1^, with typical macropulse energies of 0.06–0.8 J, and fluences of 0.7–6.1 J cm^−2^. The experiment is operated at twice the IR laser frequency, and mass spectra with and without irradiation are recorded to correct for the fluctuations during cluster production. IRMPD spectra are obtained by calculating the depletion yield *Y*(*ν*) at frequency *ν*, defined as:
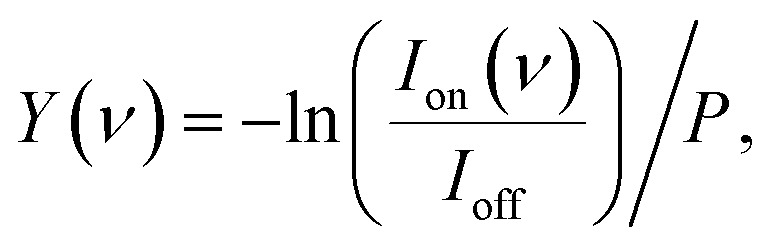
where *I*_on_(*ν*) is the ion intensity of the irradiated complex, *I*_off_ the ion intensity without irradiation, and *P* the macropulse energy. Spectra displaying the depletion *I*_on_(*ν*)/*I*_off_ can be found in Fig. S1† of the ESI.

For the structural assignment of selected spectra (for Cu_6_C^−^ and Cu_7_C^−^), DFT calculations of different isomers were done using the Q-Chem 5.3 program package.^35^ Stable structures were identified using the TPSSh/def2-TZVP + D3 level of theory,^36^ as described in the ESI.† A detailed description of the search procedure is given in the ESI.† This combination of functional, basis set and dispersion correction was chosen to be able to directly compare the results with our previous work on the adsorption of CO_2_ on cationic Cu clusters,^37^ where adsorption was limited to physisorption. The accuracy of this method was carefully evaluated compared to a CCSD(T)/def2-QZVPPD benchmark.^37^ In other studies it has been found that the precise mode of physisorption found in these calculations can markedly be influenced by the choice of dispersion correction.^38^ However, since the interaction for the species under study here is significantly stronger, as seen by the activated and dissociated products discussed below, we believe that these influences are not decisive here. This is confirmed by further DFT based computations with D3 or D4 and without dispersion correction (Table S1 in the ESI†).

Only the lowest spin multiplicities were considered in the calculations, so doublet for Cu_6_C^−^, and singlet for Cu_7_C^−^. Harmonic vibrational frequencies of these structures are convoluted with 20 cm^−1^ Gaussian line shape function and compared to the experimental data for final assignment. No frequency scaling has been applied for the comparison.

## Results and discussion

**Fig. 2:**
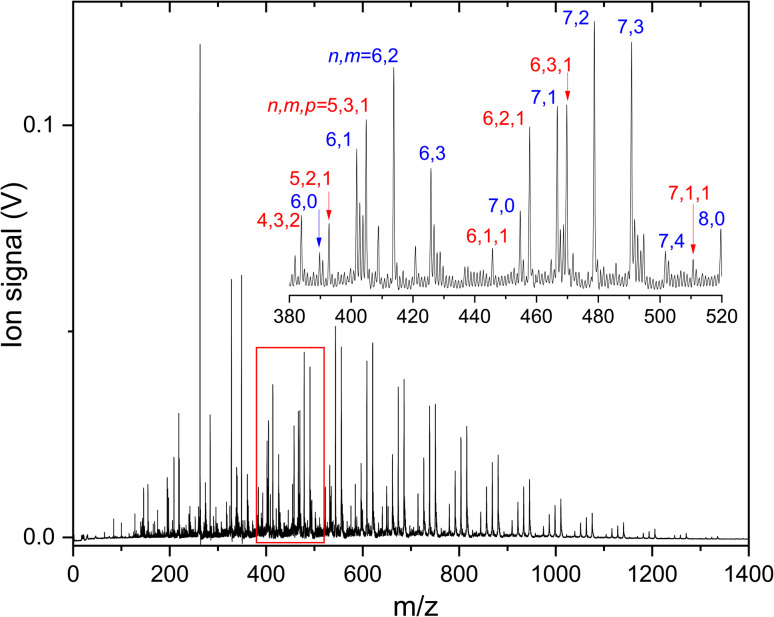
Mass spectrum of the products formed when reacting anionic Cu_*n*_C_*m*_^−^ clusters with CO_2_. Mass peaks in the inset are labeled by (*n* and *m*) or (*n*, *m*, and *p*) for Cu_*n*_C_*m*_(CO_2_)_*p*_^−^.

To understand how CO_2_ binds to anionic carbon-doped copper clusters, IRMPD spectra of products formed upon reacting Cu_*n*_C_*m*_^−^ (*n* = 3–10) with different carbon loading *m* = 1–2 are recorded. A mass spectrum of all species formed is shown in Fig. 2. From this figure, one can see that for each size *n*, a distribution of Cu_*n*_C_*m*_^−^ clusters is formed, that for larger clusters (*n* ≥ 9) is essentially limited to *m* = 0–3. Pure Cu_*n*_^−^ clusters are lower in intensity than the carbon doped clusters, which we attribute to a more facile formation of a CuC^−^ seed as a nucleation core than Cu_2_^−^.

In the inset, a close-up of the mass spectrum in the region close to Cu_6_^−^ and Cu_7_^−^ is shown, with individual mass peaks corresponding to Cu_*n*_C_*m*_(CO_2_)_*p*_^−^ labeled by (*n* and *m*) or (*n*, *m* and *p*). It can be seen that the pure Cu clusters are produced in this mass spectral region too, albeit at lower intensity than the Cu_*n*_C^−^, Cu_*n*_C_2_^−^, and Cu_*n*_C_3_^−^ signals. Unfortunately, CO_2_ adsorption on the pure Cu_*n*_^−^ clusters is negligible, precluding spectroscopic characterization.

### CO_2_ activation by Cu_*n*_C^−^

**Fig. 3:**
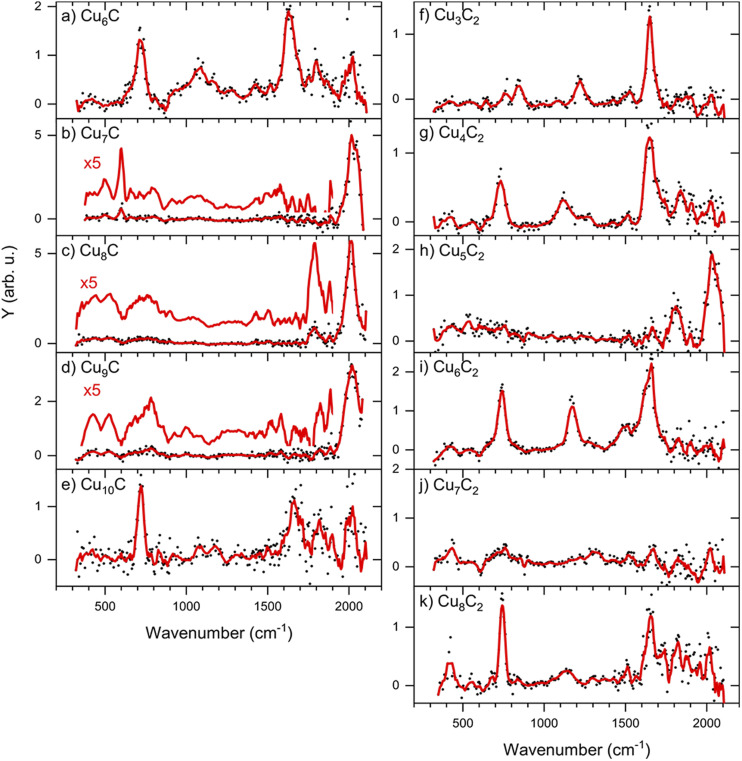
IRMPD spectra of [Cu_*n*_C·CO_2_]^−^ (left) and [Cu_*n*_C_2_·CO_2_]^−^ (right). The red lines are five-point adjacent-averages of the solid dots.

The IRMPD spectra of low-carbon loading products [Cu_*n*_C·CO_2_]^−^ (*n* = 6–10) are shown in Fig. 3 (panels a–e). The complexes with *n* = 7–9 show only one main band around 2000 cm^−1^, which is fairly close to the frequency of the free C–O stretch vibration at 2143 cm^−1^.^39^ We can thus speculate that these cluster sizes adsorb CO_2_ dissociatively, leading to the formation of CO. In contrast, the spectra of complexes with *n* = 6 and 10 are dominated by two bands around 720 and 1630 cm^−1^, with a weaker band visible around 1100 cm^−1^. Earlier, experimental IR studies of the CO_2_^−^ radical embedded in alkali halide matrices have revealed a characteristic band at 1671 cm^−1^.^40^ The presence of a band in this region in the IRMPD spectra suggests that Cu_*n*_C^−^ clusters with *n* = 6 and 10 induce a distortion of CO_2_, activating it. Bands at similar frequencies were observed when the Pt_4_^−^ ion was reacted with CO_2_, which was also concluded to lead to activation.^31^

**Fig. 4:**
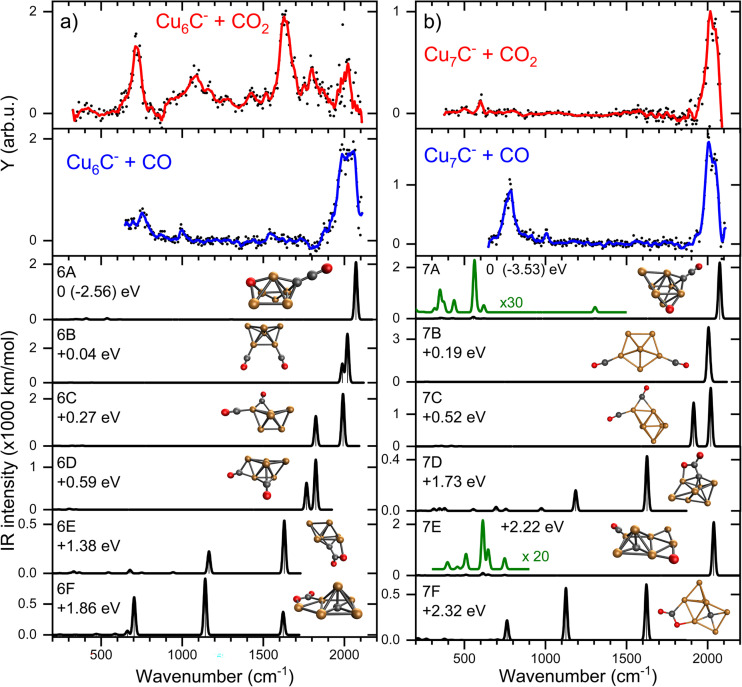
IRMPD spectra of: (a) Cu_6_C^−^, and (b) Cu_7_C^−^, reacted with CO_2_ (red) and CO (blue), and calculated spectra (black) of the lowest energy structure. All calculated spectra are convoluted with a 20 cm^−1^ FWHM Gaussian line shape function, and are accompanied by the geometrical structure (Cu, C and O atoms represented by orange, black and red spheres) and relative energy. The green curves are vertical zooms with the multiplication factors indicated. The CO_2_ binding energy is given for the lowest energy structure, relative energies for other isomers.

From here, we focus on the species [Cu_*n*_C·CO_2_]^−^ with *n* = 6 and 7 since their spectra are representative of two modes of adsorption, activated and dissociated. The suspicion that CO_2_ adsorbs dissociatively on CCu_7_^−^ can be verified by reference spectra of carbon monoxide (CO) adsorbed to Cu_7_C^−^, which is compared to the IRMPD spectrum of [Cu_7_C·CO_2_]^−^ in Fig. 4b (second panel from the top). For this, we reacted the clusters with CO and recorded IRMPD spectra in the 650–2100 cm^−1^ range for the [Cu_*n*_C·CO]^−^ (*n* = 4–10) species formed. The resulting spectra for all measured cluster sizes are dominated by a strong band around 2020 cm^−1^, characteristic for the C–O stretch. This band shows an excellent agreement with the bands observed when Cu_7_C^−^ is reacted with CO_2_ (Fig. 4b, top panel), strengthening the hypothesis that CO_2_ adsorbs dissociatively on Cu_*n*_C^−^ (*n* = 7–9). In contrast, the mismatch between spectra for [Cu_6_C·CO_2_]^−^ and [Cu_6_C·CO]^−^ (Fig. 4a) suggests that CO_2_ adsorbs predominantly molecularly on Cu_6_C^−^ and, by extension, also on Cu_10_C^−^.

**Fig. 5:**
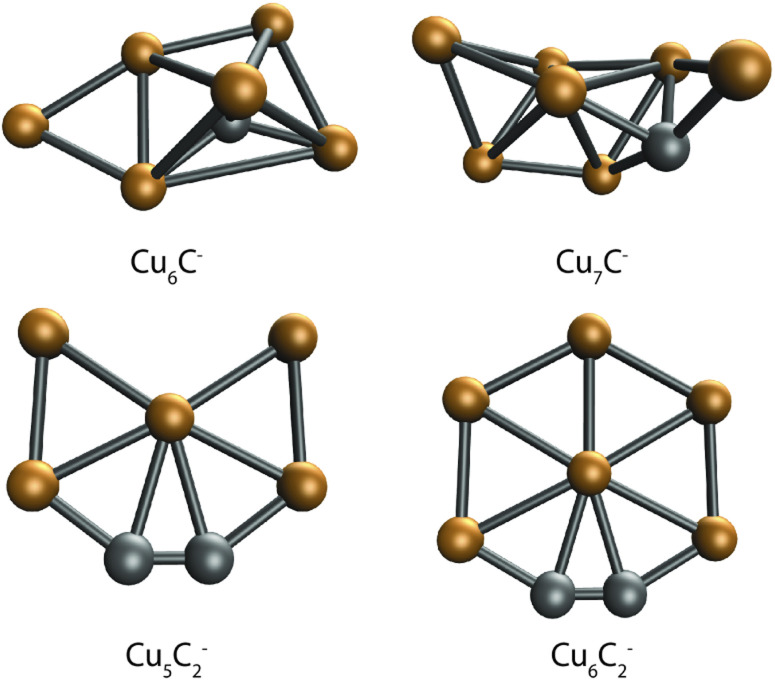
Lowest energy structures of the single C-doped clusters Cu_6_C^−^ and Cu_7_C^−^, and double C-doped clusters Cu_5_C_2_^−^ and Cu_6_C_2_^−^.

DFT calculations were performed to further interpret these observations. For this, we first searched for bare Cu_*n*_C_*m*_^−^ clusters. In our global optimization routine, we have identified the lowest energy structures shown in Fig. 5. The lowest energy structures found are not unlike those predicted for the pure clusters. Cu_6_C^−^ is similar to the predicted Cu_6_^−^ octahedron,^41^ with the C substituting one of the Cu atoms in the octahedron, and the sixth Cu atom bound to one of the Cu–Cu edges. Cu_7_C^−^ looks like the boat structure proposed for Cu_6_^−^,^42^ with a C bound on a hollow site, and the last Cu atom bound both to the Cu–Cu edge and to the C atom. Interestingly, the structures for Cu_5_C_2_^−^ and Cu_6_C_2_^−^ are planar and are similar to the structure predicted for pure Cu_5_^−^, a 2D trapezoid.^41,42^ In both clusters, the C atoms are bound together in a C_2_ unit that forms one corner of a planar hexagon for Cu_6_C_2_^−^, with one Cu atom missing for Cu_5_C_2_^−^.

Using the structures found for the bare clusters, a search was done for structures of the reaction product of Cu_6_C^−^ and Cu_7_C^−^. An extensive search has identified over sixty stable structures for each species. Many of these structures have a similar binding motif of C and O atoms to the cluster leading to only minor differences in IR spectra, which are indistinguishable in the currently applied experimental method. Therefore, the structures were grouped, first using their spectral properties only. Visual inspection of all structures per group allowed to conclude that they all shared similar binding motifs. For the CO_2_ reaction products with Cu_6_C^−^, sixteen unique spectral patterns were identified, for Cu_7_C^−^ only nine. The spectra of the lowest energy representative from each group are compared to the experimental data in Fig. S2.† A selection of the most promising candidates is shown in Fig. 4 (black traces).

In the search, structures are found both with dissociated and molecular CO_2_, where structures with CO_2_ dissociated are generally lowest in energy. The spectra of structures with dissociated CO_2_ are dominated by high-frequency bands originating from the C–O stretch vibration(s), with frequencies ranging from 1800–2100 cm^−1^, depending on the binding site of the CO. Spectra of clusters with molecularly bound CO_2_ have multiple bands typically at the lower frequencies associated with the CO_2_ bending mode, and the C–O_bound_ and C–O_free_ stretch.

For both *n* = 6 and 7, the largest number of structures found is with CO_2_ dissociatively adsorbed, where CO is bound in an on-top configuration (μ^1^) to a Cu atom, and the O eliminated from CO_2_ bound to another Cu; an example is structure 7E. However, this binding motif is not the energetically most favorable lying at least 1.78 (2.19) eV higher in energy for *n* = 6 (7) than the lowest energy structure. We attribute this simply to a computational sampling effect, where the probability to find a Cu atom in a Cu_6_C^−^ cluster is six times higher than finding a C atom.

For *n* = 6, the spectra of structures from this most populated group are very similar to that of structure 6A, the lowest energy structure, whereas this has a different structural motif. In structure 6A, the cluster structure contains a Cu_5_ square pyramid, with the sixth Cu bridge-bound (μ^2^) to one of the edges, and the C bound to a hollow (μ^3^) Cu_3_ site. Of the dissociated CO_2_, the CO is μ^1^-bound to the cluster’s C atom, forming a linear C–C–O moiety, and the O to a hollow Cu site. The spectrum has its main band, the CO stretch vibration, close to 2100 cm^−1^, or blue-shifted from the observed band frequency by about 60 cm^−1^. The same motif of CO binding to the lone C is found for the lowest energy group for *n* = 7 (*e.g.* structure 7A). 7A is a Cu_6_ octahedron capped by the seventh Cu and the C, both in μ^3^ configuration. The CO_2_ is dissociated with the single O capping a third octahedron plane, and the CO bound to the C dopant.

For both *n* = 6 and 7 the second group (*e.g.* 6B and 7B) is formed by structures where CO_2_ dissociation leads to an O atom migrating to the C dopant atom, forming two CO molecules that are μ^1^-bound to a Cu. For 6B this is in the form of a Cu_6_ boat structure with the CO molecules attached to the bow and the stern, while for 7B it is a Cu_7_ pentagonal bipyramid. The frequencies of both CO stretch vibrations are close to each other resulting in one merged vibrational band, which is found at frequencies of about 2000 cm^−1^, slightly lower than that for the C–C–O group in 6A and 7A. Group 3 (*e.g.* 6C/7C) is characterized by two CO molecules, with one μ^1^- and the other μ^2^-bound. In 7C, the same capped octahedron is recognized, with the bridge-bound CO close to the on-top CO. In this case, two distinct C–O stretch bands are seen, one around 2000 cm^−1^ for the μ^1^ bound CO, and one around 1900 cm^−1^ for the μ^2^ bound CO. For *n* = 6, group 4 has CO molecules bound in μ^2^ and μ^3^ configurations, with again clearly two distinct bands, now at 1800 and 1900 cm^−1^, consistent with a weakening of the C–O bond when it coordinates to multiple Cu atoms. An interesting motif found is the linking of two CO molecules resulting in an O–C–C–O chain. However, for both cluster sizes, this last group is lowly populated, relatively high in energy, and presents no match for the experimental spectrum.

Structures with molecularly bound CO_2_ are typically much higher in energy (>1.4 eV) for both *n* = 6 and 7. The structures found are always di-σ-bound *via* the C and the O. Formation of a C–C bond is favorable resulting in the preferential binding of the CO_2_ carbon atom to the cluster’s carbon atom, with one of the O atoms binding to a Cu atom. The lowest energy structures displaying this binding motif are 6E and 7D.

Structures are assigned based on the comparison between calculated and experimental spectra. The IRMPD spectrum of [Cu_6_C·CO_2_]^−^ shows two major bands at 718 and 1630 cm^−1^ with weaker bands at 1094, 1426, 1802, and 2016 cm^−1^. This number of bands cannot be explained by a single structure. The major band at 718 cm^−1^ agrees best with the CO_2_ bending mode predicted at 705 cm^−1^ for structure 6F, where CO_2_ is intact, but bent as a result of charge transfer. The CO_2_ molecule is bound with a C and the O to two neighboring Cu atoms in a bidentate bridging configuration. Two more intense bands are predicted at 1141 and 1620 cm^−1^ offering a good match for the experimental bands at 1094 and 1630 cm^−1^. These correspond to the C–O stretching vibrations involving the bound and free O atoms, respectively. Although the band frequencies of 6F match the experiment very well, the relative intensities are less convincing. The experimental band at 718 cm^−1^ has the highest intensity, even though it does not dominate any of the calculated spectra. We do not have a good explanation for this observation; we can speculate that it is related to the excitation mechanism in IRMPD spectroscopy, which may cause discrepancies with calculated intensities.

The search for low energy structures for Cu_6_C^−^ yielded a Cu_5_ square pyramid structure shown in Fig. 5, with the C atom fourfold coordinated to the base, and an additional Cu atom μ^2^-bound on one of the edges. In structure 6F, this structure is retained, making this a likely entrance complex. Despite the higher energy of structure 6F (+1.86 eV relative to the lowest energy structure) it is the easiest formed. The formation of other more stable isomers necessarily proceeds *via* either C–O bond cleavage or structural rearrangement of the cluster. Both processes require additional energy to overcome a barrier associated with the transition state.

The depletion spectrum of [Cu_6_C·CO_2_]^−^ (Fig. S1†) does not rule out a mixture of different isomers present in the molecular beam, since none of the bands reach 100% depletion. The enhanced intensity of the 1630 cm^−1^ experimental band could then potentially be explained by the presence of an isomer like 6E with predicted bands at 1163 and 1629 cm^−1^. Structure 6E appears also relatively easily formed as an entrance complex, where CO_2_ also binds in a bidentate bridging configuration, now with the C to the cluster’s carbon atom. However, the bare cluster’s bridging Cu is now bound on a hollow site.

The dominant experimental bands can thus be explained by molecularly adsorbed CO_2_, but minor bands above 1750 cm^−1^ more likely originate from the carbonyl C–O stretch, resulting from CO_2_ dissociation. The band at 1802 cm^−1^ might be due to structure 6C’s 1825 cm^−1^ predicted mode but could also be assigned to the doublet from structure 6D at 1764 and 1825 cm^−1^, which could have merged into one band due to band broadening. The 2016 cm^−1^ band could be the second band of structure 6C at 1990 cm^−1^ or 6A and 6B’s bands at 2069 and 2018 cm^−1^, respectively. The offset for 6A makes 6B, 6C and 6D the more likely candidates to explain the high-frequency bands.

All in all, it can be concluded that CO_2_ binds mostly molecularly to Cu_6_C^−^, but in an activated form *via* the C atom either to C or to Cu atoms. Dissociation may occur, but it is certainly not the dominant motif, which is underlined by the significantly higher predicted IR intensities of the carbonyl C–O stretch modes.

This dominance of molecular binding is in sharp contrast to what happens after adsorption of CO_2_ on Cu_7_C^−^. The IRMPD spectrum of [Cu_7_C·CO_2_]^−^ has one dominant band at 2033 cm^−1^ and a low-intensity band at 598 cm^−1^. From a comparison with low-energy isomers, it is quite clear that CO_2_ is dissociated since no bands associated with activated complexes (7D and 7F) are present in the experimental spectrum. Structure 7C has two CO binding motifs, and its spectrum has separate bands at 1915 (μ^2^, Cu–Cu) and 2017 cm^−1^ (μ^1^, Cu). Because this does not match the experimental spectrum, we must look for structures with only μ^1^ binding. Spectra calculated for such structures (7A, 7B, and 7E) are dominated by one band, depending on the adsorption site of CO, at 2078 (μ^1^ to C), 2017 (twice μ^1^ to Cu) and 2038 cm^−1^ (μ^1^ to Cu), respectively. Each of them could explain the main experimental band at 2033 cm^−1^. The low-intensity band at 598 cm^−1^ could be due to the weak bands around 558 and 600 cm^−1^ for structures 7A and 7E, respectively. These bands are more than twenty times lower in intensity than the C–O stretch. We therefore assign the spectrum to either 7A or 7E, corresponding to structures where the CO formed from dissociating CO_2_ is μ^1^-bound either to the cluster’s C or Cu atom, and O bound separately to the cluster. Both structures appear not easily formed, because the lowest energy structure for Cu_7_C^−^ (Fig. 5) requires substantial re-arrangement.

### CO_2_ activation by Cu_*n*_C_2_^−^

If CO_2_ is reacted with Cu clusters containing a second carbon, the picture changes. The spectra for the [Cu_*n*_C_2_·CO_2_]^−^ (*n* = 3–8) species are presented in Fig. 3 (right panels). Due to a different production efficiency for clusters with two C atoms, a slightly different range of *n* is presented. In contrast to the Cu_*n*_C^−^ species, where only [Cu_6_C·CO_2_]^−^ and [Cu_10_C·CO_2_]^−^ show molecular adsorption, all spectra except that for [Cu_5_C_2_·CO_2_]^−^ show bands around 740, 1150, and 1650 cm^−1^ indicating activated, but molecular adsorption of CO_2_. For all these species, bands above 1800 cm^−1^, attributed to dissociative CO_2_ adsorption, have disappeared, suggesting that only molecularly bound CO_2_ complexes are left. Only the spectrum of [Cu_5_C_2_·CO_2_]^−^ shows an intense band at 2034 cm^−1^, suggesting dissociative adsorption. From this, it can be concluded that, overall, the addition of the second C atom reduces the cluster reactivity.

Again, we selected two cluster sizes for DFT calculations, [Cu_5_C_2_·CO_2_]^−^ and [Cu_6_C_2_·CO_2_]^−^. From the glancing overview in Fig. 3 they are representative for dissociative and molecular binding of CO_2_. The comparison of the spectra for these structures with spectra of the clusters reacted with CO confirms this conclusion, because the 2034 cm^−1^ band for [Cu_5_C_2_·CO_2_]^−^ perfectly matches that observed for [Cu_5_C_2_·CO]^−^. In contrast, the spectrum for [Cu_6_C_2_·CO]^−^ does not show any counterpart for the bands dominating the spectrum of [Cu_6_C_2_·CO_2_]^−^.

**Fig. 6:**
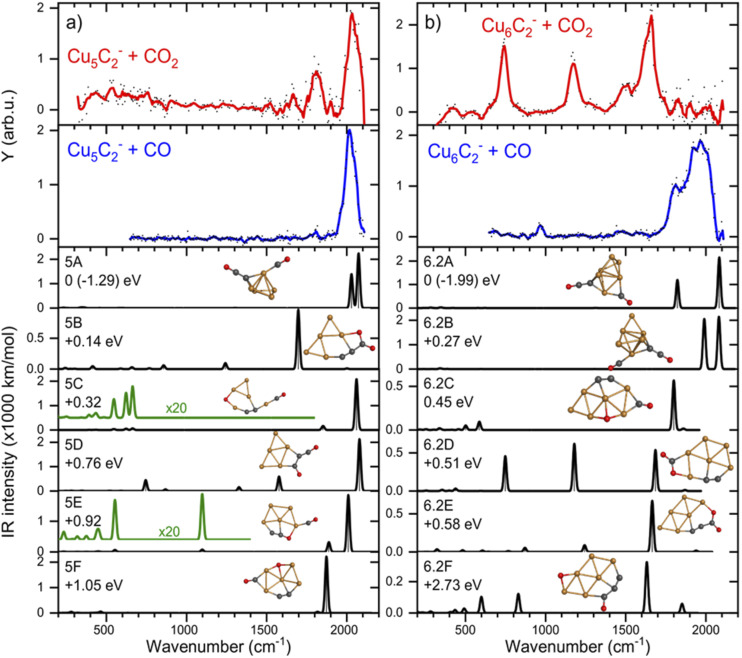
IRMPD spectra of (a) Cu_5_C_2_^−^ and (b) Cu_6_C_2_^−^ reacted with CO_2_ (red) and CO (blue), and calculated spectra (black) of the lowest energy structure. For details, see caption Fig. 4.

The calculated structures were again categorized following their spectral and adsorption motifs, as illustrated in Fig. 6. The full list of the lowest energy structures of the identified groups can be found in Fig. S3.† Interestingly, for higher carbon loading, the most common motif found is still the dissociation of CO_2_ to CO and O (5C and 6.2C), both bound to Cu atoms. The lowest energy structures 5A and 6.2A are formed by the adsorption of CO to one of the cluster’s C atoms and O to the other, forming a C–C–O and a CO moiety. These structures are comparable to the CO motif of Cu_6_C^−^ and Cu_7_C^−^, suggesting it is overall more favorable for CO_2_ to dissociate and bind to the C atoms of the cluster, rather than to Cu.

The experimental spectrum of [Cu_5_C_2_·CO_2_]^−^ is dominated by the strong band at 2035 cm^−1^ and has two more bands at 1667 and 1810 cm^−1^. The maximum depletion observed (∼45%) allows room for assignment to multiple isomers. All structures shown could potentially be present in the molecular beam since they all have bands that might overlap with the experimentally detected. The experimental band at 2035 cm^−1^ could be explained either by structure 5A with two close by bands predicted at 2077 and 2027 cm^−1^, or by structures 5C, 5D, and 5E with bands at 2063, 2078, and 2012 cm^−1^, respectively. The second band, at 1810 cm^−1^, could be explained by the main band of structure 5F at 1865 cm^−1^, or by the minor bands predicted for structures 5C and 5E. The only serious contender for the weakest band observed at 1667 cm^−1^ is structure 5B with the band at 1698 cm^−1^. All except for 5B, have CO_2_ adsorbed dissociatively on the cluster. 5B has molecularly adsorbed CO_2_ bound with the one of the C atoms attached to one of the cluster’s C atoms, and the O to a Cu.

If we compare the structures shown in Fig. 6a to the lowest energy structure for Cu_5_C_2_^−^, predicted to have a 2D, wheel-like structure, it appears that all structures apart from 5A could be formed without all too large structural re-arrangements of the original cluster: all retain a planar structure with the two C atoms of the bare clusters close together. Only the formation of 5A upon CO_2_ adsorption requires a major structural re-arrangement.

In contrast to the spectrum of [Cu_5_C_2_·CO_2_]^−^, the spectrum of [Cu_6_C_2_·CO_2_]^−^ does not exhibit any bands above 1780 cm^−1^, which is indicative that CO_2_ is adsorbed molecularly. Therefore, structures 6.2A, 6.2B and 6.2C can be ruled out since they all have high-intensity bands at 1800 cm^−1^ or above. Three of the four experimental bands (742, 1173, and 1648 cm^−1^) could quite well be explained by isomer 6.2D with bands at 748, 1179, and 1685 cm^−1^. The band at 1648 cm^−1^ could also be explained by the 1648 cm^−1^ band of structure 6.2E or by structure 6.2F’s 1634 cm^−1^ band, but these structures lack intense bands at lower frequencies. Only the experimental band at 1484 cm^−1^, a shoulder of the 1648 cm^−1^ band, cannot be explained by isomer 6.2D. Interestingly, it appears quite similar to the weaker band at 1426 cm^−1^ in the [Cu_6_C·CO_2_]^−^ spectrum, which may have a similar origin. Another parallel drawn from the spectrum of [Cu_6_C·CO_2_]^−^ is that the relative intensities of the middle band are not reflecting the calculated ratios.

### Reactive potential energy surface

It is now of interest to understand what factors could determine dissociation or lack thereof. For this, we attempted to calculate reaction pathways for two representative clusters. The complexity associated with reconstructing the reactive pathway of CO_2_ adsorption and subsequent activation over carbon-doped Cu clusters is enormous. First, as experimental evidence for the structures of the bare clusters is unknown, the starting point is ill-defined. Nevertheless, using the minima found in Fig. 5, we calculated a reaction pathway for CO_2_ dissociation. It is clear that the structural phase-space for CO_2_ initial adsorption is large, and the pathways to dissociation are plenty. We therefore limit ourselves to potential pathways that could illuminate whether there could exist a difference in barrier for even or odd numbers of Cu atoms.

**Fig. 7:**
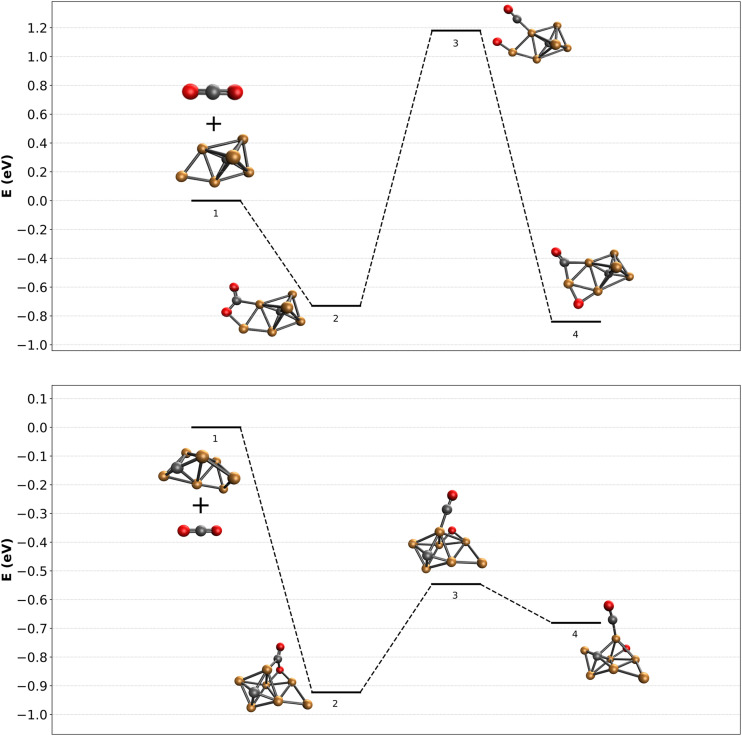
Reactive potential energy surface for CO_2_ dissociation over the lowest energy structures found for Cu_6_C^−^ and Cu_7_C^−^, calculated at the TPSSh/def2-TZVPD level of theory. Energies (in eV) are given with respect to the bare clusters and free CO_2_.

In the top panel of Fig. 7, the adsorption and subsequent dissociation of CO_2_ over Cu_6_C^−^ is shown. Adsorption leads to the formation of structure 6F, already thought to be an entrance complex, with an adsorption energy of 0.73 eV. The CO_2_ is adsorbed *via* the C to one of the Cu atoms forming the base of the octahedron, and the O atom to the capping extra Cu atom. From here, CO is abstracted towards the base Cu atom, after which it is bound to the next Cu–Cu bridge, overall gaining little energy with respect to the entrance complex. The transition state associated with abstracting the CO is relatively high at 1.18 eV above the reactants. Such a barrier is insurmountable, certainly under the room-temperature near-thermal conditions of the reaction channel used here.

The bottom panel of Fig. 7 shows the same reaction path, now calculated for CO_2_ adsorbing onto Cu_7_C^−^. The entrance complex found is much higher in energy than anything shown in Fig. 4b, at about 2.5 eV higher than structure 7A, the lowest energy structure. The cluster is an octahedral Cu_5_C, with the sixth Cu (denoted Cu(6)) bridge-bound to a Cu–Cu edge, and the seventh to the newly formed Cu–Cu(6) edge. CO_2_ is adsorbed *via* its C atom to an octahedron Cu atom, and *via* an O atom to the other Cu–Cu(6) edge. CO abstraction then leads to an energetically still not very favorable structure with the CO bound on top of a Cu atom, and the O atom on a hollow Cu–Cu–Cu site. The initial adsorption energy of CO_2_ onto Cu_7_C^−^ is with 0.93 eV not much different from that of Cu_6_C^−^. In contrast, the dissociation reaction over Cu_7_C^−^ is much more facile than over Cu_6_C^−^, and with an energy barrier 0.55 eV lower than the energy of the reactants, this dissociation reaction is well possible.

These two reaction paths are by no means a complete description of the reactions taking place. However, they do allow to draw initial conclusions. (1) The observation of dominant molecular adsorption in the experimental spectrum of [Cu_6_C·CO_2_]^−^ is consistent with the high barrier calculated from structure 6F in Fig. 7; (2) finding a dissociation barrier below the energy of the reactants for Cu_7_C^−^ demonstrates that dissociation should occur, also consistent with the observed spectrum. It may not be this pathway, as lower barrier pathways may exist, but if an exothermic reaction with a barrier below the reactants exists, the system should find it.

So what is now the influence of the carbon atom on the dissociation propensity? We recall that for Cu_*n*_C^−^ the dominant adsorption form is dissociative and for Cu_*n*_C_2_^−^ molecular. If extra carbon atoms have a decisive influence, one would compare the outcome of the CO_2_ adsorption reaction for clusters with the same number of atoms, where Cu atoms are exchanged for C atoms. For this comparison, we evaluate the spectra in Fig. 3, comparing Cu_6_C^−^ with Cu_5_C_2_^−^, Cu_7_C^−^ with Cu_6_C_2_^−^, and so on. If anything, one sees that this comparison gives opposite outcomes: Cu_6_C^−^ molecular, Cu_5_C_2_^−^ dissociative; Cu_7_C^−^ dissociative, Cu_6_C_2_^−^ molecular; Cu_8_C^−^ dissociative, Cu_7_C_2_^−^ inconclusive; Cu_9_C^−^ dissociative, Cu_8_C_2_^−^ molecular. Then, if we compare clusters with the same number of Cu atoms: Cu_6_C^−^ and Cu_6_C_2_^−^ molecular, Cu_7_C^−^ dissociative, Cu_7_C_2_^−^ inconclusive; Cu_8_C^−^ dissociative, Cu_8_C_2_^−^ molecular. The only trend, if one may call it this: the addition of the second carbon to the cluster inhibits the dissociative adsorption for all cluster sizes except for *n* = 5. We can only speculate that the addition of an extra C atom, which has the higher electron affinity of C (2.55) compared to Cu (1.90),^43^ leads to a decrease of the charge located on the Cu atoms thereby reducing the electron donation from the cluster into the CO_2_ antibonding orbital. Of course, such a hypothesis requires a more detailed investigation, including spectroscopic characterization of CO_2_ activation by the pure Cu_*n*_^−^ clusters that was elusive in this work.

## Conclusion

IRMPD spectroscopy of the CO_2_ adsorbed on the anionic copper carbides reveals a size-dependent bonding nature. Single-carbon doped Cu clusters Cu_*n*_C^−^ with *n* = 6 and 10 show predominantly activated adsorption, but dissociative adsorption of CO_2_ for *n* = 7–9. These results were verified with control experiments of CO adsorbed on the same cluster sizes and with DFT calculations. The exact position of the C and O atoms on the clusters could not be determined precisely, since the bands for specific structures are very similar in frequency. Therefore, only the main binding motifs have been analyzed. Doubly-carbon doped Cu clusters Cu_*n*_C_2_^−^ with *n* = 3–8 show predominantly molecular adsorption, excepting Cu_5_C_2_^−^ which shows dissociative adsorption of CO_2_. DFT calculations of the dissociation reaction of CO_2_ over Cu_6_C^−^ and Cu_7_C^−^ are consistent with the observed IR spectra, showing only activation for the former and dissociation for the latter. To understand why the addition of a second C atom apparently throttles CO_2_ dissociation requires further extensive computational work, evaluating reactive pathways over other cluster compositions. We speculate that the higher electron affinity of C relative to that of Cu will reduce the capacity of Cu atoms in higher C/Cu ratio clusters to donate electron density to CO_2_, reducing the activation capabilities.

## Conflicts of interest

There are no conflicts to declare.

## Supplementary Material

FD-242-D2FD00128D-s001
