# Supplementary material for: Tuning the degree of CO2 activation by carbon doping Cun− (n = 3–10) clusters: an IR spectroscopic study
Source: Faraday Discuss. 2022 Nov 3;242:252–68. doi: 10.1039/d2fd00128d (PMC9890493; doi:10.1039/d2fd00128d)
Supplement: FD-242-D2FD00128D-s001 [file FD-242-D2FD00128D-s001.pdf]

Electronic Supplementary Material (ESI) for Faraday Discussions.
This journal is © The Royal Society of Chemistry 2022

Tuning the degree of CO_2 activation by carbon doping Cu_n^- (n=3-10) clusters: an IR spectroscopic study.

Olga V. Lushchikova,[a,b] Máté Szalay[c], Tibor Höltzl[c], and Joost M. Bakker[a,*]

Supporting Information

Contents

Infrared spectra 2
Computational details on finding lowest energy cluster structures and adsorbate geometries 5
Dispersion correction and Cartesian coordinates 6
References 10

Infrared spectra

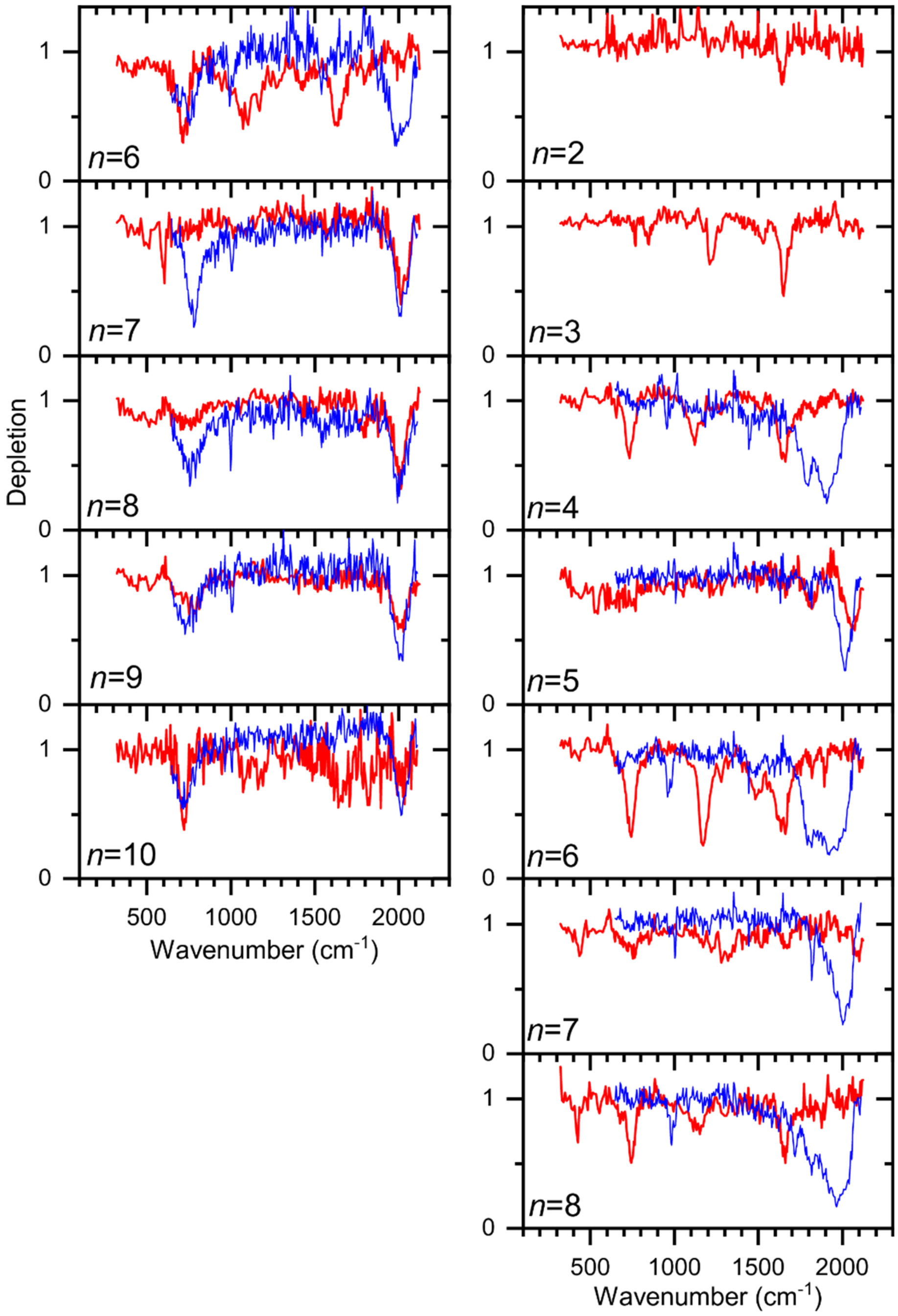

Figure S1: IR depletion spectra of Cu_nC^- (left) and $Cu_nC_2^-$ (right) reacted with CO_2 (red) and CO (blue).

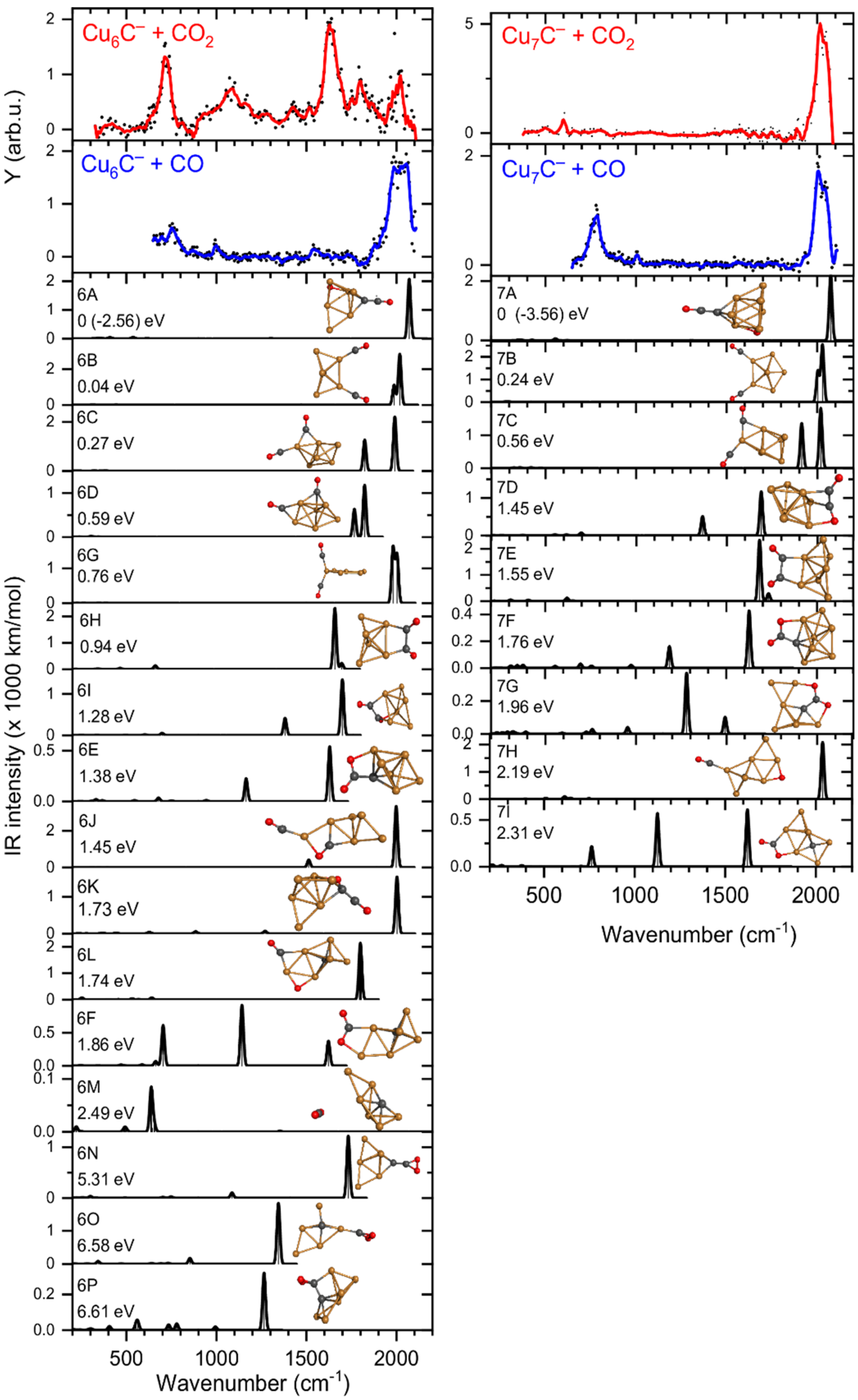

Figure S2: IRMPD spectra of: Cu_6C^- and Cu_7C^- reacted with CO_2 (red) and CO (blue), and calculated spectra (black) of the lowest energy structures per group (see main text). All calculated spectra are convoluted with a 20 cm^{-1} FWHM Gaussian line shape function, and are accompanied by the geometrical structure (copper, carbon and oxygen atoms represented by orange, black and red spheres) and relative energy. For the lowest energy structure, the energy relative to the reactants is given in parentheses.

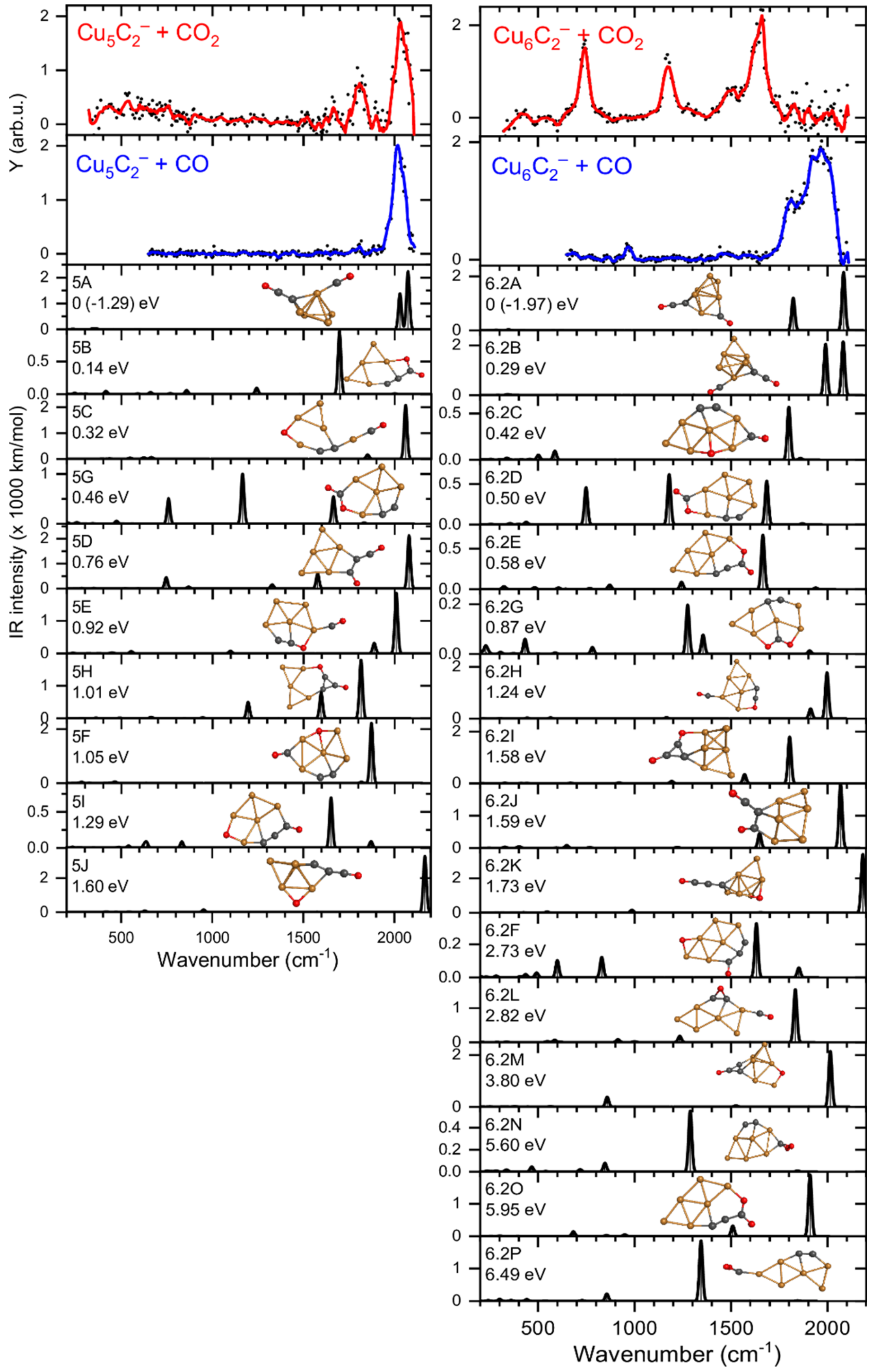

Figure S3: IRMPD spectra of: $Cu_5C_2^-$ (left) and $Cu_6C_2^-$ (right) reacted with CO_2 (red) and CO (blue), and calculated spectra (black) of the lowest energy structure per group. All calculated spectra are convoluted with a 20 cm^{-1} FWHM Gaussian line shape function, and are accompanied by the geometrical structure (copper, carbon and oxygen atoms represented by orange, black and red spheres) and relative energy. For the lowest energy structure, the energy relative to the reactants is given in parentheses.

Computational details on finding lowest energy cluster structures and adsorbate geometries

Lowest energy geometries of the bare $Cu_nC_m^-$ clusters were searched by generating several possible initial structures based on the pure copper cluster geometries, also using the capping method[i] and finally were systematically analyzed and confirmed using the CALYPSO method and software[ii,iii,iv] in conjunction with the Gaussian16[v] program code and employing the BP86/LANL2DZ method. The lowest energy cluster structures were re-optimized using the more accurate TPSSh/def2-TZVPD level of theory.

The target of the adsorption computations is not to locate the global minimum structure, but to explore the possible kinetically accessible adducts. The initial geometries for the cluster-adsorbate systems were systematically generated using our program based on the Molmod library,[vi] while the reaction paths are explored subsequently. This methodology was applied successfully in our recent works[vii,viii,ix,x.xi]. The investigated intact and dissociated CO_2 binding motifs are depicted in Figure S5.

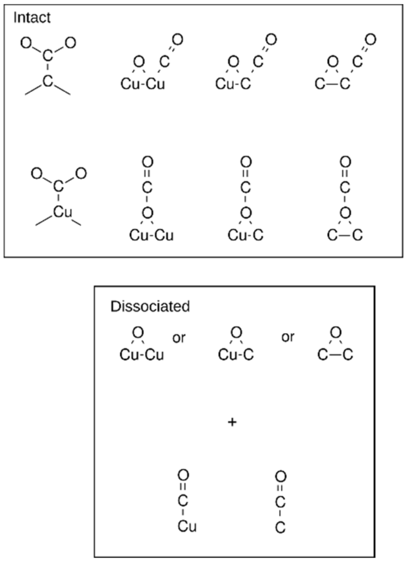

Figure S5. The different CO_2 binding motifs used in the systematic search for the intact and dissociated adsorbate structures.

Dispersion correction and Cartesian coordinates

Table S1. Reaction energies of different structures using different Grimme type dispersion corrections (Functional: TPSSh, basis: def2-TZVPD)

Structure	*No dispersion correction*	*D3xii,xiii*	*D4*[xiv,xv]
7B	**-3.20 eV**	**-3.27 eV**	**-3.32 eV**
7F	**-1.08 eV**	**-1.14 eV**	**-1.26 eV**

Table S2. Cartesian coordinates of structures in Figure 4

#	XYZ Coordinates				#	XYZ Coordinates			
6A	Cu	-0.14019	-1.41229	0.07019	7A	Cu	-1.17134	-0.73662	-1.15087
	Cu	-1.52845	0.52908	-0.46114		Cu	2.13669	1.67738	-0.16636
	Cu	1.36315	0.30117	1.24278		Cu	0.28853	-2.00534	0.44259
	Cu	-2.51118	-1.45602	0.34256		Cu	-1.24330	-0.31802	1.31892
	Cu	0.88017	0.51897	-1.12456		C	-2.11219	0.66885	-0.19721
	Cu	0.11836	2.27877	0.52110		Cu	1.31063	-0.35842	-1.10547
	C	1.75444	-0.97568	-0.23157		Cu	-0.24274	1.35737	-0.21329
	C	2.74758	-1.79812	-0.35067		Cu	1.14684	0.00293	1.41039
	O	3.66293	-2.54771	-0.46589		C	-3.25226	1.27995	-0.18431
	O	-0.44870	1.87418	-1.23960		O	-4.30511	1.83232	-0.16550
						O	0.26169	-1.91380	-1.49105
6B	Cu	0.82808	0.01958	-1.22528	7B	Cu	0.41720	0.00005	-1.24980
	Cu	-0.78657	-1.29009	0.12820		Cu	1.18402	-1.95163	-0.00163
	Cu	0.85851	0.02525	1.24873		Cu	1.13704	1.96948	-0.00025
	Cu	1.52506	-1.97227	-0.07564		Cu	-1.22704	-1.28252	-0.00026
	Cu	1.41512	2.05061	-0.08319		C	-2.75342	-2.29673	-0.00019
	Cu	-0.85602	1.24575	0.12392		Cu	0.41607	-0.00041	1.24920
	C	-2.51735	1.96276	-0.06855		Cu	-1.25714	1.24433	-0.00099
	C	-2.40532	-2.09333	-0.07594		Cu	2.55557	0.02593	0.00260
	O	-3.49791	-2.44969	-0.16639		C	-2.77880	2.26755	0.00008
	O	-3.62773	2.26188	-0.14841		O	-3.77576	2.84180	0.00121
						O	-3.76832	-2.83890	0.00300
6C	Cu	0.25629	-0.29151	1.35943	7C	Cu	-2.17168	0.05754	0.01849
	Cu	1.91029	-1.10252	-0.54182		Cu	1.72081	0.58024	-1.29989
	Cu	-0.43611	-1.64234	-0.52948		Cu	-0.19547	1.35993	-0.08881
	Cu	2.24126	0.97237	0.63271		Cu	1.91028	-1.45589	0.09130
	Cu	-1.81361	0.33442	0.07082		C	-3.22913	-1.41398	0.00322
	Cu	0.37094	0.69777	-0.85145		Cu	-0.07114	-0.70326	1.29401
	C	-1.11567	2.00951	-0.63243		Cu	-0.15154	-0.96258	-1.08409
	C	-3.50996	-0.27921	0.29414		Cu	1.82039	0.83398	1.08412
	O	-4.53833	-0.72531	0.57082		C	-2.27568	1.93664	-0.01928
	O	-1.16030	3.16793	-0.82538		O	-2.46853	3.08644	-0.03337
						O	-3.77635	-2.42701	-0.00945
6D	Cu	0.38833	-0.04987	-1.22620	7D	Cu	1.07330	1.24070	0.60595
	Cu	0.40906	-1.29538	0.81071		Cu	-0.98514	-2.17103	-0.10769
	Cu	1.21764	1.00584	0.89906		Cu	2.61919	-0.05694	-0.79387
	Cu	2.57665	-0.56354	-0.27634		Cu	-2.31043	-0.01283	-0.65590
	Cu	-1.18071	0.81306	0.57668		C	-0.94423	1.21437	0.01073
	Cu	-1.70718	-1.27432	-0.42927		Cu	0.09807	-0.13887	-1.10491
	C	0.21765	1.87529	-0.62171		Cu	-0.92728	-0.20911	1.31264
	C	-3.02663	0.18242	0.17043		Cu	1.18148	-1.25672	0.72702
	O	-4.17544	0.43064	0.10812		C	-0.83560	2.69583	-0.00251
	O	0.10597	2.97137	-1.05525		O	0.29704	3.06558	0.55237
						O	-1.67803	3.44418	-0.49777

6E	Cu	0.40987	-0.35702	-1.35557	7E	Cu	0.66491	-1.67933	-0.34508
	Cu	0.71831	-1.27068	0.80910		Cu	-2.04941	0.85545	-0.30702
	Cu	0.88786	1.33046	0.31626		Cu	2.73369	-0.34537	-0.14380
	Cu	2.63219	-0.24657	-0.36827		Cu	-0.87862	-0.59031	1.40785
	Cu	-1.40290	1.16922	-0.62374		C	-0.83692	-0.56519	-0.55074
	Cu	-0.84560	0.32565	1.63284		Cu	0.55522	0.78267	-0.14883
	C	-1.04629	-0.76957	-0.07132		Cu	-2.34213	-1.64333	-0.27145
	C	-2.47171	-1.04659	-0.40791		Cu	2.58664	2.01228	0.12113
	O	-3.03467	0.04636	-0.88797		C	-3.20655	2.23739	-0.28853
	O	-3.02586	-2.13184	-0.24112		O	-3.97855	3.08697	-0.23388
						O	2.40624	-2.13734	-0.27059
6F	Cu	-0.04024	-1.54554	-0.20714	7F	Cu	2.56174	1.00253	0.26238
	Cu	-0.89094	0.80534	-0.19917		Cu	-2.02974	-0.07642	0.05950
	Cu	1.00827	0.16006	1.39889		Cu	0.15487	0.81661	1.07223
	Cu	-2.37761	-1.13833	-0.07692		Cu	0.04837	-1.57541	0.04490
	Cu	2.46512	-0.91457	-0.34513		C	1.22323	-0.14657	-0.30073
	Cu	2.09606	1.55201	-0.42897		Cu	-0.11102	0.82775	-1.29780
	C	0.89908	0.10748	-0.54099		Cu	2.56975	-1.42914	-0.19324
	C	-2.84332	1.30107	-0.07136		Cu	-1.52787	2.31264	0.00129
	O	-3.69380	0.35098	0.08807		C	-2.48758	-2.01303	0.12617
	O	-3.04291	2.51139	-0.14199		O	-1.43979	-2.79077	0.15245
						O	-3.65153	-2.39930	0.16237

Table S3. Cartesian coordinates of structures on Figure 5

	XYZ Coordinates					XYZ Coordinates			
Cu_6C^-	Cu	-2.37172	-0.90704	0.55130	Cu_7C^-	Cu	0.08161	-1.65264	0.57266
	Cu	-0.78433	-0.38280	-1.35530		Cu	-2.12030	-0.72800	0.75262
	Cu	0.83070	1.09638	0.04441		Cu	-0.87763	-0.30246	-1.22355
	Cu	-1.61678	1.41943	0.22885		Cu	1.61849	-0.53700	-1.13439
	Cu	0.99836	-1.23144	0.30498		Cu	0.19313	0.86079	0.56081
	Cu	3.07056	0.04491	0.11172		Cu	-2.02603	1.59668	-0.22185
	C	-0.61285	-0.19065	0.55117		Cu	2.80255	0.86651	0.53693
						C	1.58617	-0.50211	0.75770
$Cu_5C_2^-$	Cu	0.00019	-0.07097	0.00415	$Cu_6C_2^-$	Cu	2.16603	-0.97491	0.01373
	Cu	-2.15481	0.98804	-0.00070		Cu	-2.24986	1.39903	0.00872
	Cu	2.15513	0.98781	-0.00180		Cu	-2.16353	-0.97825	-0.00626
	Cu	-2.04696	-1.38639	-0.00144		Cu	0.00241	-2.09481	-0.00394
	Cu	2.04636	-1.38681	-0.00035		Cu	-0.00022	0.27402	-0.00294
	C	-0.63248	2.09826	-0.00025		Cu	2.24620	1.40249	-0.01004
	C	0.63293	2.09856	0.00090		C	-0.63523	2.34965	0.00601
						C	0.63019	2.35040	-0.00250

Table S4. Cartesian coordinates of structures Figure 6

#	XYZ Coordinates				#	XYZ Coordinates			
5A	Cu	-0.42851	-0.89254	-1.08187	6.2A	Cu	-1.32727	-1.34845	-0.24422
	Cu	1.07945	-1.15495	0.97793		Cu	2.14123	0.42346	-1.23996
	Cu	1.85545	-0.25902	-1.20396		Cu	0.96626	-0.94713	0.43340
	C	-1.80222	0.29636	-0.30346		C	-3.16467	0.98457	-0.57609
	Cu	-1.22731	-0.72169	1.27859		Cu	-0.17508	0.84644	-0.96670
	C	-2.93415	0.75697	-0.70821		C	-2.02363	0.44572	-0.29863
	Cu	0.06283	1.05491	0.18464		Cu	-0.86561	0.62275	1.33472
	C	1.12574	2.46475	0.57984		Cu	1.40296	1.29856	1.01800
	O	1.83617	3.33420	0.82756		C	0.00757	-2.67131	0.09878
	O	-3.99261	1.18038	-1.06680		O	0.33360	-3.80419	0.18434
						O	-4.21457	1.48829	-0.81765
5B	Cu	-0.93721	-1.53716	-0.00101	6.2B	Cu	-0.50173	-0.52754	1.49176
	Cu	0.50279	2.49423	-0.00085		Cu	2.95950	0.47256	-0.39278
	Cu	-3.10315	-0.59331	0.00072		Cu	-1.35820	1.65923	1.19035
	C	2.07016	-1.70538	-0.00165		C	-0.86695	-3.10899	0.02167
	Cu	1.23831	0.24167	-0.00083		Cu	1.18003	-1.07473	-0.33280
	C	0.86300	-2.01688	-0.00163		C	-0.56055	-1.85481	0.03125
	Cu	-1.15791	0.82840	0.00131		Cu	-1.22241	-0.15234	-0.78178
	C	3.42954	-1.10332	-0.00002		Cu	0.70390	1.26805	0.03542
	O	4.46209	-1.76385	0.00263		C	-2.10891	0.77535	-2.06858
	O	3.29813	0.18540	0.00225		O	-2.57011	1.43875	-2.89117
						O	-1.16148	-4.26138	0.01608
5C	Cu	2.60718	-0.77769	0.00124	6.2C	Cu	2.50613	1.13088	-0.21447
	Cu	-2.15249	0.86998	-0.00645		Cu	-3.60345	-0.37535	-0.49483
	Cu	-0.22788	2.28322	0.00586		Cu	1.95970	-1.31493	0.02923
	C	-0.09626	-2.14501	0.00696		C	-0.30394	2.25341	0.16011
	Cu	-1.86627	-1.60778	-0.00211		Cu	-1.90490	1.23989	-0.04743
	C	1.14526	-1.88747	0.00975		C	0.95868	2.20360	0.10102
	Cu	0.08638	-0.05859	0.00916		Cu	0.28274	0.34102	0.71251
	C	4.11120	0.19409	-0.01059		Cu	-1.48589	-1.20851	0.10530
	O	5.05629	0.84396	-0.01878		C	3.59209	-0.48133	-0.51940
	O	-3.29655	-0.53581	-0.01374		O	4.70305	-0.74714	-0.85754
						O	0.25241	-1.55672	0.72386
5D	Cu	-0.23719	-1.51012	-0.00064	6.2D	Cu	2.73894	-1.36046	-0.00219
	Cu	-0.33071	2.74817	-0.00345		Cu	-1.67229	0.92730	-0.00642
	Cu	-2.67600	-1.32262	-0.00068		Cu	2.62623	1.02083	-0.00235
	C	2.44930	-0.55158	0.00023		C	-0.11518	-2.23089	-0.00018
	Cu	1.03645	0.80500	0.00456		Cu	-1.85141	-1.61394	0.00198
	C	1.70505	-1.81287	-0.00169		C	1.14424	-2.34502	-0.00080
	Cu	-1.38292	0.63604	0.00191		Cu	0.49841	-0.24592	0.00365
	C	3.73319	-0.32099	-0.00076		Cu	0.42283	2.10677	0.00558
	O	4.86282	0.02037	-0.00188		C	-3.57648	0.30530	-0.00155
	O	2.23665	-2.92344	-0.00260		O	-4.45009	1.15390	-0.00408
						O	-3.65420	-0.97628	0.00502
5E	Cu	2.02819	-0.64373	-0.00058	6.2E	Cu	2.65152	0.90014	0.01655
	Cu	-1.99819	1.51532	-0.00037		Cu	-3.59446	-0.37640	0.02095
	Cu	0.85667	1.65030	-0.00013		Cu	0.60568	2.24435	-0.00043
	C	-1.22253	-2.15782	0.00032		C	0.29484	-2.08381	-0.01087
	Cu	-2.65437	-0.87867	-0.00178		Cu	-1.52068	-1.48800	-0.00363
	C	0.05554	-2.23269	0.00038		C	1.53487	-1.86882	-0.00913
	Cu	-0.41425	-0.23103	0.00340		Cu	0.49236	-0.06623	-0.02739
	C	3.72638	0.02278	-0.00179		Cu	-1.62548	1.03829	-0.00970
	O	4.68582	0.66167	-0.00098		C	3.02064	-1.90948	0.00566
	O	1.30423	-2.50505	-0.00019		O	3.62889	-0.76211	0.01732
						O	3.57592	-3.00535	0.00668
5F	Cu	2.57937	-0.93682	0.19907	6.2F	Cu	3.15550	0.22313	-0.01310
	Cu	-1.53000	1.21514	0.00678		Cu	-3.22762	0.18497	-0.02052
	Cu	2.00316	1.43779	0.17631		Cu	1.37289	1.89448	0.00444
	C	-0.19249	-2.22509	-0.09037		C	1.73321	-2.32997	-0.01629
	Cu	-1.81833	-1.23959	0.05762		Cu	-1.36135	-1.28070	-0.00082
	C	1.07322	-2.11866	-0.05906		C	2.80579	-1.65635	-0.02591
	Cu	0.37358	-0.18991	-0.44134		Cu	0.85279	-0.41118	0.01119

	C	-3.14477	0.43971	0.23344		Cu	-0.96511	1.17670	0.01605
	O	-4.28255	0.16272	0.34560		C	0.30748	-2.49548	0.00803
	O	0.15238	1.72633	-0.40292		O	-0.37747	-3.51299	0.03233
						O	-2.63061	1.89503	0.00329

Table S5 Cartesian coordinates of structures on Figure 7a

#	XYZ Coordinates				#	XYZ Coordinates			
1	Cu	-0.89612	0.82356	-0.21241	2	Cu	-0.02170	-1.56212	-0.21384
	Cu	0.98567	0.13193	1.41062		Cu	-0.89612	0.82356	-0.21241
	Cu	-2.35053	-1.14173	-0.07201		Cu	0.98567	0.13193	1.41062
	Cu	2.48558	-0.87101	-0.35856		Cu	-2.35053	-1.14173	-0.07201
	Cu	2.07823	1.55700	-0.41305		Cu	2.48558	-0.87101	-0.35856
	C	0.88199	0.10695	-0.52312		Cu	2.07823	1.55700	-0.41305
						C	0.88199	0.10695	-0.52312
						C	-2.86525	1.28318	-0.07267
						O	-3.70372	0.31630	0.03702
						O	-3.07791	2.49224	-0.10034
3	Cu	-0.25302	-1.67641	-0.14596	4	Cu	-0.35762	-1.49103	-0.20346
	Cu	-0.73477	0.79173	-0.15227		Cu	-0.67916	1.00064	-0.08628
	Cu	1.15557	-0.10833	1.42574		Cu	1.12716	-0.15681	1.35575
	Cu	-2.53528	-1.03443	-0.14101		Cu	-2.61448	-0.49879	-0.07996
	Cu	2.74276	-0.67564	-0.48206		Cu	2.67188	-0.72244	-0.51056
	Cu	1.74748	1.52396	-0.42773		Cu	1.71554	1.56565	-0.30098
	C	0.90235	-0.20557	-0.47447		C	0.89884	-0.12702	-0.59456
	C	-2.06496	2.07118	0.06073		C	-2.73927	1.35711	-0.04424
	O	-4.00058	-0.03691	-0.17029		O	-3.32998	2.38282	-0.03053
	O	-2.82239	2.91204	0.20247		O	-2.04421	-2.20780	-0.12302

Table S6. Cartesian coordinates of structures on Figure 7b

#	XYZ Coordinates				#	XYZ Coordinates			
1	Cu	0.08169	-1.65272	0.57281	2	Cu	-2.70242	0.02841	0.29896
	Cu	-2.12020	-0.72764	0.75311		Cu	-0.63974	0.32676	-1.19401
	Cu	-0.87763	-0.30290	-1.22374		Cu	0.63786	-1.17086	0.52553
	Cu	1.61888	-0.53728	-1.13415		Cu	-1.48513	-1.89672	-0.49630
	Cu	0.19322	0.86103	0.56053		Cu	1.72482	0.75805	-0.40483
	Cu	-2.02643	1.59638	-0.22226		Cu	3.02758	-1.09929	0.21322
	Cu	2.80237	0.86690	0.53694		Cu	-0.54343	1.04203	1.07268
	C	1.58582	-0.50158	0.75771		C	-1.19239	-0.83174	1.02601
						C	0.14633	2.63684	0.10343
						O	0.21813	3.84216	0.25134
						O	0.49556	2.09606	-1.15374
3	Cu	-2.68731	0.20932	0.28100	4	Cu	-2.88919	-0.12552	0.17562
	Cu	-0.75674	-0.02406	-1.33488		Cu	-0.69652	-0.27279	-1.28742
	Cu	0.61733	-1.02329	0.67603		Cu	0.69274	-0.82854	0.80132
	Cu	-1.59439	-1.92615	-0.01631		Cu	-1.41404	-2.06415	0.30835
	Cu	1.75171	0.45174	-0.86304		Cu	1.84009	0.36497	-0.96808
	Cu	3.01775	-1.04532	0.46277		Cu	3.09294	-0.75653	0.69112
	Cu	-0.39627	1.30331	0.63615		Cu	-0.22401	1.71779	-0.10147
	C	-1.18538	-0.49422	1.14099		C	-1.11877	-0.23524	0.67409
	C	0.15526	3.03962	0.80291		C	-0.49110	3.03381	1.08562
	O	0.53076	4.11940	0.76746		O	-0.71793	3.78745	1.92189
	O	0.41553	1.41889	-1.65159		O	0.46801	1.23592	-1.86214

References

[i] Höltzl, T., Veldeman, N., De Haeck, J., Veszpremi, T., Lievens, P., & Nguyen, M. T. (2009). Growth Mechanism and Chemical Bonding in Scandium-Doped Copper Clusters: Experimental and Theoretical Study in Concert. *Chemistry–A European Journal*, *15*(16), 3970-3982.

[ii] Wang, Y., Lv, J., Zhu, L., & Ma, Y. (2012). CALYPSO: A method for crystal structure prediction. *Computer Physics Communications*, *183*(10), 2063-2070.

[iii] Wang, Y., Lv, J., Zhu, L., & Ma, Y. (2010). Crystal structure prediction via particle-swarm optimization. *Physical Review B*, *82*(9), 094116.

[iv] Lv, J., Wang, Y., Zhu, L., & Ma, Y. (2012). Particle-swarm structure prediction on clusters. *The Journal of chemical physics*, *137*(8), 084104.

[v] Gaussian 16, Revision C.01, M. J. Frisch, G. W. Trucks, H. B. Schlegel, G. E. Scuseria, M. A. Robb, J. R. Cheeseman, G. Scalmani, V. Barone, G. A. Petersson, H. Nakatsuji, X. Li, M. Caricato, A. V. Marenich, J. Bloino, B. G. Janesko, R. Gomperts, B. Mennucci, H. P. Hratchian, J. V. Ortiz, A. F. Izmaylov, J. L. Sonnenberg, D. Williams-Young, F. Ding, F. Lipparini, F. Egidi, J. Goings, B. Peng, A. Petrone, T. Henderson, D. Ranasinghe, V. G. Zakrzewski, J. Gao, N. Rega, G. Zheng, W. Liang, M. Hada, M. Ehara, K. Toyota, R. Fukuda, J. Hasegawa, M. Ishida, T. Nakajima, Y. Honda, O. Kitao, H. Nakai, T. Vreven, K. Throssell, J. A. Montgomery, Jr., J. E. Peralta, F. Ogliaro, M. J. Bearpark, J. J. Heyd, E. N. Brothers, K. N. Kudin, V. N. Staroverov, T. A. Keith, R. Kobayashi, J. Normand, K. Raghavachari, A. P. Rendell, J. C. Burant, S. S. Iyengar, J. Tomasi, M. Cossi, J. M. Millam, M. Klene, C. Adamo, R. Cammi, J. W. Ochterski, R. L. Martin, K. Morokuma, O. Farkas, J. B. Foresman, and D. J. Fox, Gaussian, Inc., Wallingford CT, 2016.

[vi] Verstraelen, T: Molmod software Library, https://molmod.ugent.be/software

[vii] Lushchikova, O. V., Szalay, M., Tahmasbi, H., Juurlink, L. B., Meyer, J., Höltzl, T., & Bakker, J. M. (2021). IR spectroscopic characterization of the co-adsorption of CO 2 and H 2 onto cationic Cu n+ clusters. *Physical Chemistry Chemical Physics*, *23*(47), 26661-26673.

[viii] Szalay, M., Buzsáki, D., Barabás, J., Faragó, E., Janssens, E., Nyulászi, L., & Höltzl, T. (2021). Screening of transition metal doped copper clusters for CO 2 activation. *Physical Chemistry Chemical Physics*, *23*(38), 21738-21747.

[ix] Barabás, J., Ferrari, P., Kaydashev, V., Vanbuel, J., Janssens, E., & Höltzl, T. (2021). The effect of size, charge state and composition on the binding of propene to yttrium-doped gold clusters. *RSC advances*, *11*(47), 29186-29195.

[x] Barabás, J., Vanbuel, J., Ferrari, P., Janssens, E., & Höltzl, T. (2019). Non-covalent Interactions and Charge Transfer between Propene and Neutral Yttrium-Doped and Pure Gold Clusters. *Chemistry–A European Journal*, *25*(69), 15795-15804.

[xi] Hou, G. L., Faragó, E., Buzsáki, D., Nyulászi, L., Höltzl, T., & Janssens, E. (2021). Observation of the reaction intermediates of methanol dehydrogenation by cationic vanadium clusters. *Angewandte Chemie International Edition*, *60*(9), 4756-4763.

[xii] Grimme, S., Antony, J., Ehrlich, S., & Krieg, H. (2010). A consistent and accurate ab initio parametrization of density functional dispersion correction (DFT-D) for the 94 elements H-Pu. The Journal of chemical physics, 132(15), 154104.

[xiii] Witte, J., Mardirossian, N., Neaton, J. B., & Head-Gordon, M. (2017). Assessing DFT-D3 damping functions across widely used density functionals: Can we do better?. Journal of chemical theory and computation, 13(5), 2043-2052.

[xiv] Caldeweyher, E., Bannwarth, C., & Grimme, S. (2017). Extension of the D3 dispersion coefficient model. The Journal of chemical physics, 147(3), 034112.

[xv] Caldeweyher, E., Ehlert, S., Hansen, A., Neugebauer, H., Spicher, S., Bannwarth, C., & Grimme, S. (2019). A generally applicable atomic-charge dependent London dispersion correction. The Journal of chemical physics, 150(15), 154122.
